# Impulsivity trait mediates the relationship between white matter integrity of prefrontal–striatal circuits and the severity of dependence in alcoholism

**DOI:** 10.3389/fpsyt.2022.985948

**Published:** 2022-09-07

**Authors:** Fei Wu, Ping Dong, Guowei Wu, Jiahui Deng, Zhaojun Ni, Xuejiao Gao, Peng Li, Bing Li, Junliang Yuan, Hongqiang Sun

**Affiliations:** ^1^Peking University Sixth Hospital, Peking University Institute of Mental Health, NHC Key Laboratory of Mental Health (Peking University), National Clinical Research Center for Mental Disorders (Peking University Sixth Hospital), Peking University, Beijing, China; ^2^Chinese Institute for Brain Research, Beijing, China; ^3^Department of Neurology, Peking University Sixth Hospital, Peking University Institute of Mental Health, NHC Key Laboratory of Mental Health (Peking University), National Clinical Research Center for Mental Disorders (Peking University Sixth Hospital), Peking University, Beijing, China

**Keywords:** alcohol dependence, impulsivity, systematic striatal circuits, probabilistic tractography, diffusion tensor imaging

## Abstract

**Background:**

Alcohol dependence (AD) remains one of the major public health concerns. Impulsivity plays a central role in the transfer from recreational alcohol use to dependence and relapse. White matter dysfunction has been implicated in alcohol addiction behaviors and impulsivity. However, little is known about the role of systematic striatal structural connections underlying the mechanism of impulsive traits in AD.

**Methods:**

In our study, we used seed-based classification by probabilistic tractography with five target masks of striatal circuits to explore the differences in white matter integrity (fractional anisotropy, FA) in AD male patients (*N* = 51) and healthy controls (*N* = 27). We mainly explored the correlation between FA of the striatal circuits and impulsive traits (Barratt Impulsiveness Scale, BIS-11), and the mediation role of impulsivity in white matter integrity and the severity of alcohol dependence.

**Results:**

Compared with healthy controls, AD showed much lower FA in the left and right striatum–supplementary motor area (SMA) and left striatum–amygdala. We also found the decreased FA of right striatum-vlPFC was correlated with higher impulsivity. Besides, the relationship between reduced FA of right striatum-vlPFC and severity of dependence could be mediated by impulsivity.

**Conclusion:**

In our study, we found disrupted white matter integrity in systematic striatal circuits in AD and the decreased FA of right striatum-vlPFC was correlated with higher impulsivity in AD. Our main findings provide evidence for reduced white matter integrity of systematic striatal circuits and the underlying mechanisms of impulsivity in male AD individuals.

## Introduction

Alcohol dependence (AD) is a chronic relapsing disease characterized by an impaired ability to control alcohol consumption despite adverse consequences (1, 2). As the most prevalent drug of all substance use disorders, alcohol use disorder (AUD) is one of the leading global causes of disease burden and substantial health loss. A high proportion of disease burden is attributable to complex outcomes, including unintentional injuries, cancer, cardiovascular and cerebrovascular diseases, cirrhosis, and suicide (3, 4). The risk of mortality is positively associated with the level of alcohol consumption, which is potentially harmful at any level (5).

It has been reported the prognosis of AD was unfavorable after patients achieve abstinence, which attributed to a high risk of relapse (6). Impulsivity plays a central role in the transfer from recreational alcohol use to alcohol dependence and relapse. Impulsivity increases the risk of relapse which may be related to impaired inhibitory control and enhanced motivation after cue exposure, leading more serious state of alcohol dependence (7). Impulsivity is a complex structure including impulsive traits and impulsive behavior, and there are some relationships between subtypes of impulsivity, with evidence of go/no-go performance and Barratt Impulsiveness Scale scores (8). Previous studies found multiple subtypes of impulsivity were associated with a common biological factor: low dopamine D2 receptor function in striatum (9). The striatum is a complex structure interconnected with the cerebral cortex, projecting in “loops” to executive, motor, and limbic regions of the brain (10). The impulsive system includes amygdala–striatum regions, reflective system which contains the prefrontal cortex, and insula cortex which plays a key role in modulating the dynamics between these two systems (11). It was reported that individuals with more severe alcohol dependence exhibit weaker frontal (e.g., the insula, medial prefrontal cortex, and anterior cingulate) functional connectivity with the striatum, and these networks are important for response inhibition. These findings suggest the fronto-striatal pathway underlying inhibition control is weakened in AD (12). Thus, a network of interacting brain regions and associated circuits has been shown to mediate impulsivity and inhibition control behaviors of AD, including striatum, dorsolateral prefrontal cortex (dlPFC), ventrolateral prefrontal cortex (vlPFC), supplementary motor area (SMA), insular, and amygdala (9, 13, 14).

White matter (WM) structures efficiently propagate neural signals between spatially distinct cortical regions and then improve brain connectivity (15, 16). WM damage is one of the characteristic injuries of AD (17, 18). The clinical features and pathological behaviors in alcoholics are related to the integrity of WM (19, 20), especially more evidence of the structural damage on prefrontal–striatal circuits was found in AD. The reduced FA value of the orbitofrontal cortex and nucleus accumbens (OFC-NAcc) network suggests structural network alterations in AD. The increased OFC-NAcc functional connection is associated with craving (19). Besides, frontal reduced WM integrity as predictors of the alcohol treatment outcome (21). Diffusion tensor imaging (DTI) is a quantitative non-invasive method to assess the integrity of WM mainly by using the value of fractional anisotropy (FA). The reduced FA can be attributed to degradation of both myelin sheaths and axonal membranes (22, 23). Tract-based spatial statistics (TBSS) is generally used for voxel-wise analysis of whole-brain white matter (24). In spite that TBSS has advantages over other methods such as voxel-based morphometry, however, TBSS also has limited anatomical specificity and lack of information between different regions of interest (ROI) (25, 26). As a result, we will utilize the probability tractography to detect the white matter differences within specific tracts in our study.

Most prior studies have employed TBSS analysis to investigate the relationship between FA and impulsivity (27, 28). In addition, the exact relationship between WM integrity and impulsivity is still inconsistent in AD. Our previous TBSS analysis found there is no relationship between FA of whole-brain skeleton and impulsive trait (BIS-11) (27). Another tractography-based segmentation study found higher impulsivity level was associated with lower FA in corpus callosum extending to the orbitofrontal cortex in AD (29). So far, it is still unclear whether abnormal FA of systematic striatal circuits is associated with impulsivity and the severity of dependence in AD. The aim of our study was to investigate the integrity of striatal circuits by using seed-based classification with DTI probabilistic tractography and its relationship with impulsivity and severity of alcohol dependence in AD.

The aim of our study was to investigate the integrity of striatal circuits by using seed-based classification with DTI probabilistic tractography and its relationship with impulsive trait and severity of alcohol dependence in AD.

## Materials and methods

### Subjects

A case–control, cross-sectional study was conducted in the Peking University Institute of Mental Health. Diagnostic and Statistical Manual of Mental Disorders-IV (DSM-IV) criteria were assessed with the mini-international neuropsychiatric interview. Fifty-one males meeting the DSM-IV criteria for AD were recruited, and the age range was 31 and 59 years old. All AD participants were patients in hospital of the Peking University **Sixth** hospital. AD patients had finished acute withdrawal with abstinence for at least **2** weeks. In the study sample, the average Michigan Alcoholism Screening Test (MAST) score of all 51 subjects was 13.51 ± 4.15 (1–40) (see Table 1). Inclusion criteria included male, aged 30–60, right-handed, meeting the DSM-IV diagnostic criteria for AD, and the score of the Clinic Institute Alcohol Withdrawal Syndrome Scale (CIWA-AR) was less than seven. Exclusion criteria included any other Axis I psychiatric disorder or other substance use disorder (except for nicotine), any systemic or neurological disease, claustrophobia, or any other contraindication for magnetic resonance examination (MRI). Meanwhile, a total number of age- and gender-matched 27 participants were involved as healthy control (HC). The control participants were recruited from the community and, based on structured interview, had never met DSM-IV criteria for AD or any other DSM-IV Axis I disorder. All participants completed informed consent.

**Table 1:** Demographics and clinical traits of AD and HC individuals.

	**AD**	**HC**	**Z/t/*χ^2^***	***p*-value**
	**(*n* = 51)**	**(*n* = 27)**		
Age (years)	41.00 (37.00, 49.00)	37.00 (33.00, 47.00)	−1.136	0.256
Education (years)	12.00 (9.00, 15.00)	12.00 (10.00, 16.00)	−0.712	0.476
Marital status			0.052	0.819
Married	44 (86.3)	12 (81.5)		
Others	7 (13.7)	5 (18.5)		
Age at first use (years)	18.00 (16.00, 20.00)	21.00 (18.25, 36.50)	−2.366	0.018^*^
Duration of dependence	6.58 ± 4.40	–		
Mean ethanol intake (g/d)	188.35 ± 100.91	–		
Duration of abstinence (d)	16.00 (14.00, 29.00)	–		
CIWA-Ar	1.80 ± 1.47	–		
MAST	13.51 ± 4.15	0.33 ± 1.18	−6.436	<0.001^***^
Smoking			40.074	<0.001^***^
Yes	46 (90.2)	4 (18.5)		
No	5 (9.8)	17 (81.5)		
FTND	6.00 (5.00, 7.25)	2.50 (0.50, 5.25)	−5.897	<0.001^***^
BIS-11 (Total)	53.00 (42.00, 65.00)	45.00 (41.00, 55.00)	−1.404	0.160

CIWA-AR, Alcohol Withdrawal Syndrome Scale; MAST, Michigan alcoholism screening test; FTND, Fagerström test for nicotine dependence; BIS-11, Barratt impulsiveness scale, 11th version. Indexes indicate a statistically significant difference between the two groups with a *p*-value < 0.05.

* and *** indicated group differences at the *p* < 0.05 and 0.001 values, respectively.

### Procedure

Our study consisted of **two** sessions. General and clinical data were collected in the **first** session including sociodemographic, alcohol use-related, and clinical characteristics. Trait impulsivity was measured by the BIS-11, which is **one** of the most widely used self-report measures of impulsivity trait. It includes 30-item self-administered questionnaire (30). MAST was used as a severity index for alcohol dependence in our study. MAST is a rapid, reliable, inexpensive measure of a severity index for alcoholism (31). CIWA-Ar was used for clinical quantitation of the severity of the alcohol withdrawal syndrome in the baseline measurement (32). A neuropsychological assessment of cue reactivity paradigm was made. Participants were exposed to visual, olfactory, and proprioceptive stimuli associated with the beverage in alcohol cue trial. Alcohol-related cue reactivity was assessed by subjective responses (visual analog scales of craving, C-VAS, and Alcohol Urge Questionnaire, AUQ) and physiological responses (heart rate, HR; systolic blood pressure, SBP; and diastolic blood pressure, DBP) before and immediately after alcohol exposure. All subjects were requested to evaluate the intensity of their craving of alcohol consumption on a 100-point Visual Analog Scale (VAS) ranging from zero (not at all) to 100 (extremely high). The AUQ was also used to evaluate the level of intensity of alcohol craving (33). FTND is a widely used test for assessing physical nicotine dependence (34). FTND was used to assess dependence of nicotine in this study. After 15 min of rest, a **second** session was finished by the MRI scan.

### MRI data acquisition

The experiment was carried out on the GE Discovery MR750 3.0T at the Peking University Sixth Hospital. The three-dimensional (3D) T1-weighted images were acquired using an MPRAGE pulse sequence with a voxel size of 1 × 1 × 1 mm^3^ [repetition time (TR) = 6.7 ms; echo time (TE) = 2.9 ms; data matrix = 240 × 240 mm^2^; slices = 170; field of view (FOV) = 240 × 240 mm^2^]. DTI data were collected through a single-shot EPI sequence with a voxel size 2 × 2 × 2 mm^3^ (TR = 8,900 ms, TE = 92 ms, data matrix = 120 × 120, FOV = 240 × 240 mm^2^, slice =72). To improve the signal-to-noise ratio, 64 repeats of the 32 non-collinear directions (b = 1,000 s/mm^2^) were applied with eight acquisitions without diffusion weighting (b = 0 s/mm^2^).

### TBSS analysis

A voxel-wise analysis (TBSS) along whole-brain WM tracts was applied to detect the difference on the diffusivity parameters (such as FA and MD) among AD and HC. After preprocessing, we visually checked all FA maps of subjects for data quality insurance. The FA image was first normalized into to the standard MNI (Montreal Neurological Institute) space by using the fnirt command implemented in FSL. Then, an averaged FA map was created for skeleton generation step. Those voxels that lower on 0.2 in FA value were excluded based on prior research (24). Finally, FA image of each subject was projected onto the FA skeleton image. A permutation *t*-test was used to test FA differences between groups in FSL (FSL randomize procedure). The threshold-free cluster enhancement at *P*-value < 0.05 (5,000 permutations) was used to control the Type I error induced by multiple comparisons across voxels.

### Striatum structural connectivity analysis

Masks of target regions, including four cortical regions [dlPFC (3,7/4, 8), vlPFC (7,15/10, 16), SMA (19/20), and insula (29/30)] and one subcortical region [amygdala (41/42)], and mask of seed region [striatum (71,73/72,74)] were extracted from the standard Anatomical Automatic Labeling (AAL) template (https://www.gin.cnrs.fr/en/tools/aal/) and then were applied to individual brain space through a two-step normalization of ANTs tool (Advanced Normalization Tools, https://github.com/ANTsX/ANTs) to build white matter connectivity within striatum (35).

### Probabilistic tractography

Preprocessing steps of diffusion images in FDT (FMRIB's Diffusion Toolbox, https://fsl.fmrib.ox.ac.uk/fsl/fslwiki/FDT) included image quality check, 8 b0 images averaging, eddy current correction for distortions coming from EPI artifacts and motion correction, a rotation of b-vector, and tensor fitting, to eventually obtain FA maps.

Probabilistic tractography method based on seed was used to tract fibers between striatum and target regions. Calculation of fiber orientation distribution for each voxel through BEDPOSTX (GPU version) (36) was then used in fiber tracking from seeds to targets (one seed and one target at a time) by the probtrackx algorithm (GPU version) with the following parameters: streamlines = 5,000; step length = 0.5 mm; curvature threshold = 0.29 (35, 37). Tractography was separately conducted for each hemisphere. Voxels in fiber that had streamlines below the threshold of 5% of the maximum streamlines from seed to target were excluded by fslstat function (37). Eventually, we kept those voxels beyond threshold as a fiber mask in individual diffusion space to compute the mean FA as FA measurement of the fiber.

### Statistical analysis

The statistical analyses were conducted using SPSS, version 24 (IBM, Armonk, NY). Independent *t*-tests, Kruskal–Wallis test, and chi-square test were used to compare demographic and clinical characteristics between AD and HC. Then, we further employed correlations analysis to test the relationship between BIS-11 and clinical and behavioral data (such as alcohol use features and cue reactivity) in AD. We included age and education as covariates in DTI data. TBSS and seed-based probabilistic fiber tracking method were used to compare the FA of whole-brain skeleton and striatal circuits in AD and HC. After that, we employed partial correlations analysis to test whether the FA of striatal circuits and abnormal FA values of TBSS were associated with BIS-11 in AD and HC. To investigate the relationship between white matter integrity of striatal circuits and severity of alcohol dependence (MAST) (38), we performed a single-level mediation analysis with impulsivity as a mediator. We tested whether the association between FA of right striatum-vlPFC (X) and severity of alcohol dependence (Y), measured by the MAST, was mediated by self-reported impulsivity (M), measured by the BIS-11. This tests whether FA reduction is associated with impulsivity, which in turn leads to severer drinking behavior. A false discovery rate (FDR) method was used to control the rate of type I error caused by multi-comparisons in all statistics.

## Results

### Demographics, clinical characteristics, and behavior data

There are no significant differences in age, education, and marital status between the two groups. The onset of alcohol use in AD patients is earlier (Z = −2.366, *p* < 0.05), and the score of MAST is much higher than HC [t (76) = −6.436, *p* < 0.001]. The nicotine use in AD is much more serious than HC [t (76) = −5.897, *p* < 0.001] (Table 1).

The correlation analyses were made between impulsivity and alcohol-related cue reactivity (subjective responses, e.g., C-VAS and AUQ, physiological responses e.g., HR and blood pressure) and alcohol use features (mean ethanol intake per day and age at first use) in AD. Significant positive correlations were found between the BIS-11 and cue-induced craving changes (C-VAS: *r* = 0.332, *p* = 0.021; AUQ: *r* = 0.302, *p* = 0.031). The mean alcohol intake per day is also significantly positively correlated with impulsivity (*r* = 0.353, *p* = 0.011) (Supplementary Table 1). However, the correlations between BIS-11 and cue-induced craving variations were not significant after FDR correction. There is no significant relationship in HC.

### Integrity difference between AD and HC in the whole-brain WM skeleton and correlation with impulsivity

**Figure 1:**
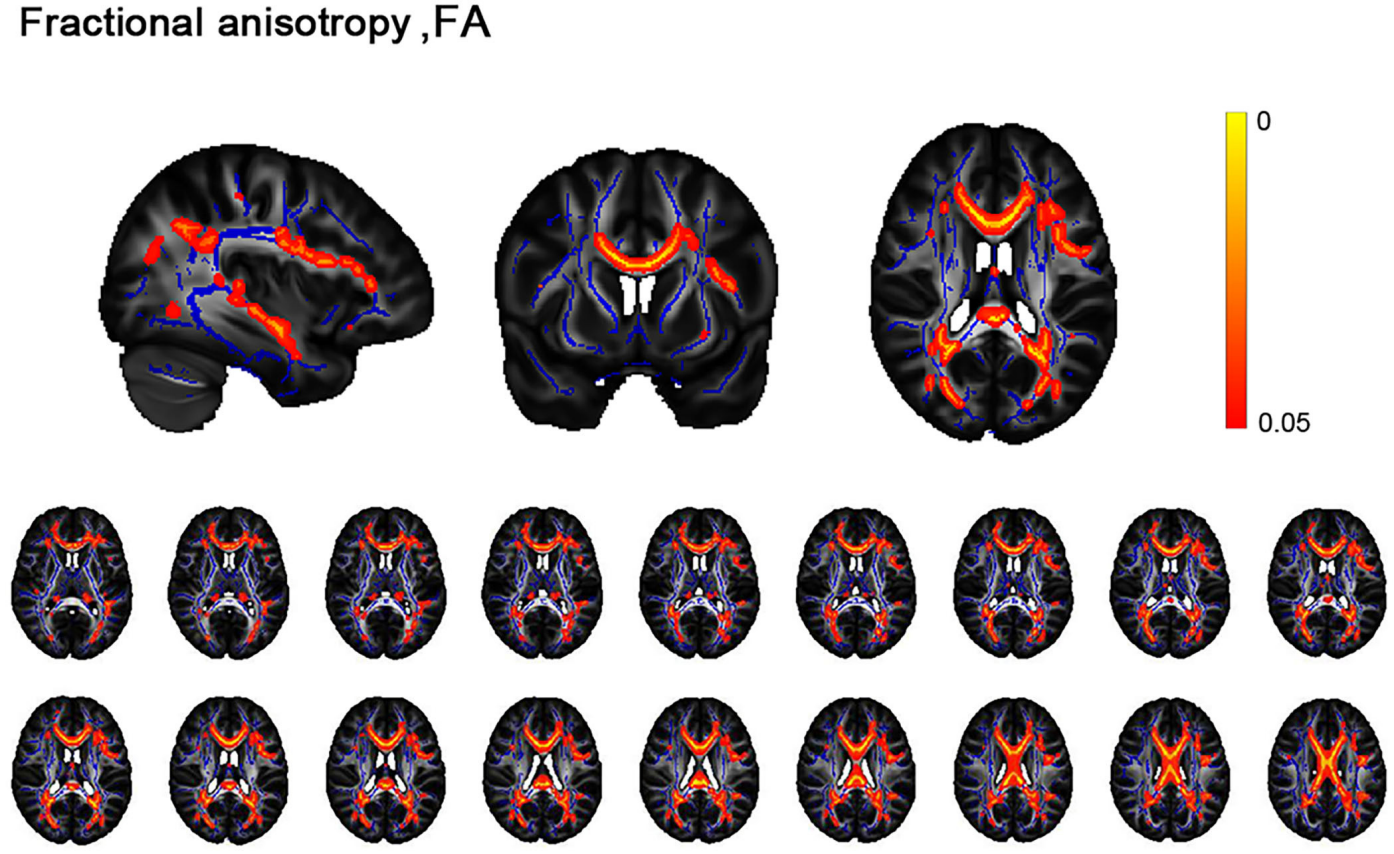
Differences between alcohol dependence (AD) cohorts and healthy controls (HC) in the white matter skeleton. Tract-based statistical analysis shows cross-sectional differences in the white matter skeleton between controls and the AD cohort undergoing diffusion tensor imaging for fractional anisotropy. AD had widespread microstructural abnormalities, namely reduced FA compared with controls at *p* < 0.05.

Patients with AD had reduced FA of widespread microstructural compared with HC at *p* < 0.05, corrected for FDR (Figure 1), mainly located in the forceps minor, forceps major, left superior longitudinal fasciculus, and right inferior frontal-occipital fasciculus (Supplementary Table 2).

**Figure 2:**
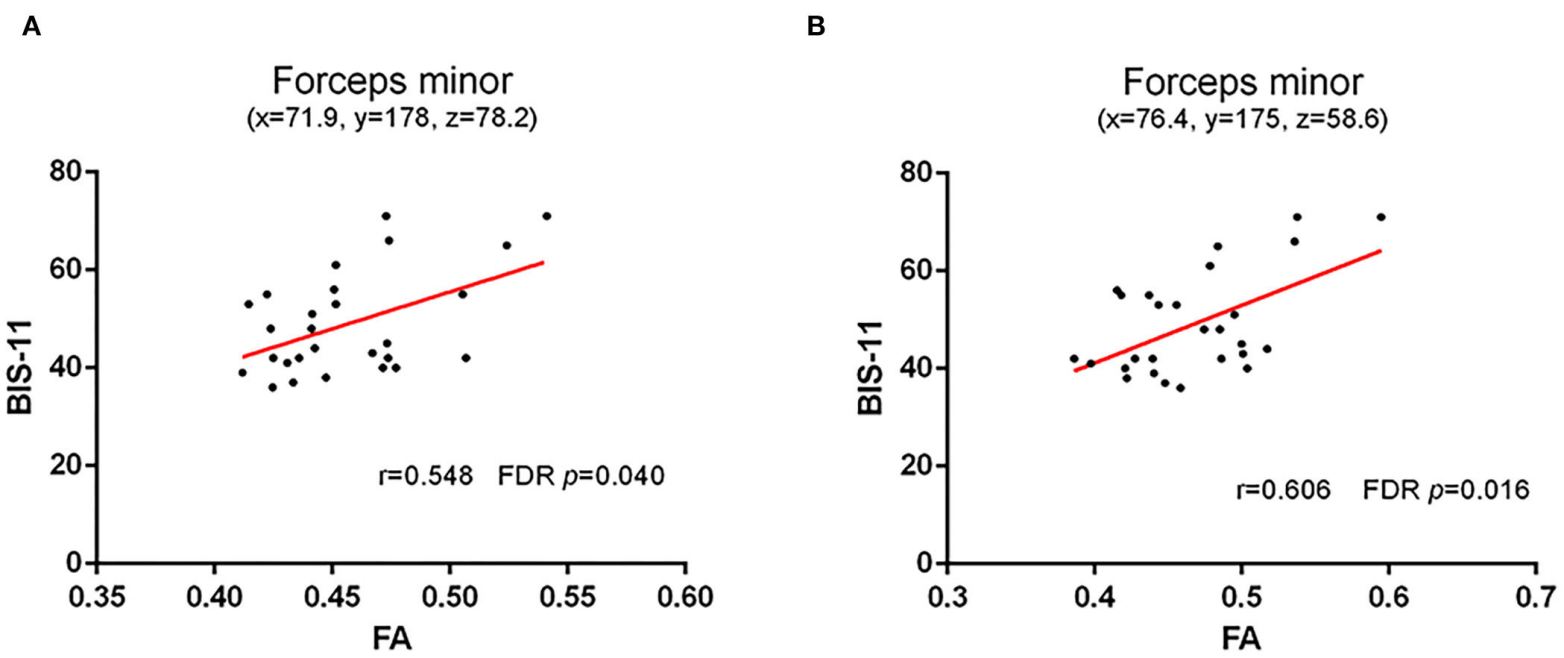
Correlation between FA of WM skeleton and BIS-11 in HC. **(A)** A positive correlation between FA of forceps minor (cluster with local maxima coordinates: x = 71.9, y = 178, z = 78.2) with the impulsive trait (BIS-11); and **(B)** A positive correlation between FA of forceps minor (cluster with local maxima coordinates: y = 175, z = 58.6) with the impulsive trait (BIS-11).

Considering the impact factors of white matter integrity, age, education, and nicotine use were controlled in partial correlation analysis. All tracts with a significant difference between the two groups in the above TBSS analysis were included in the partial correlations with impulsivity (BIS-11). Our results found a positive correlation between FA of forceps minor (cluster with local maxima coordinates: x = 71.9, y = 178, z = 78.2, R = 0.548, *p* < 0.05 and cluster with local maxima coordinates: x = 76.4, y = 175, z = 58.6, R = 0.606, *p* < 0.05) with BIS-11 in HC (Figure 2; Supplementary Table 3). However, there is no correlation between FA of WM skeleton and BIS-11 in AD (Supplementary Table 4).

### WM integrity difference in the striatal circuits and the correlation with impulsivity

**Figure 3:**
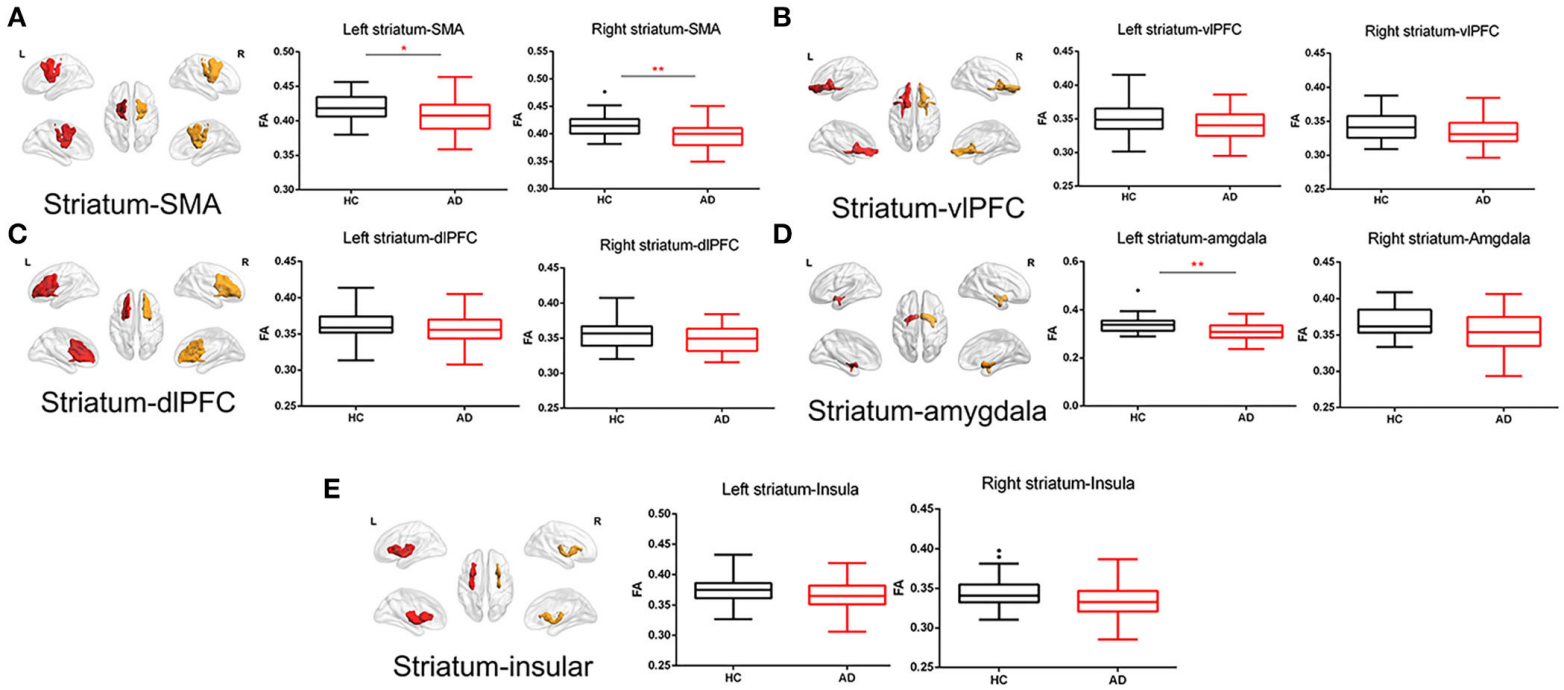
White matter integrity of striatal circuit structural connection comparisons between AD and HC. **(A)** The comparisons of FA of left and right striatum–supplementary motor area between two groups; **(B)** The comparisons of FA of left and right striatum–ventrolateral prefrontal cortex between two groups; **(C)** The comparisons of FA of left and right striatum–dorsolateral prefrontal cortex between two groups; **(D)** The comparisons of FA of left and right striatum–amygdala between two groups; and **(E)** The comparisons of FA of left and right striatum–insular between two groups. The * and ** symbols indicate the group differences at the *p* < 0.05 and *p* < 0.01 respectively.

Compared with HC, AD showed much weaker white matter integrity in the left (*t* = 0.218, *p* = 0.011, FDR *p* = 0.037) and right (*t* = −3.251, *p* = 0.002, FDR *p* = 0.010) striatum–SMA, left striatum–amygdala (*t* = −3.492, *p* = 0.001, FDR *p* = 0.010), and right striatum–insular (*t* = −2.181, *p* = 0.032, FDR *p* = 0.080) (Figure 3).

**Figure 4:**
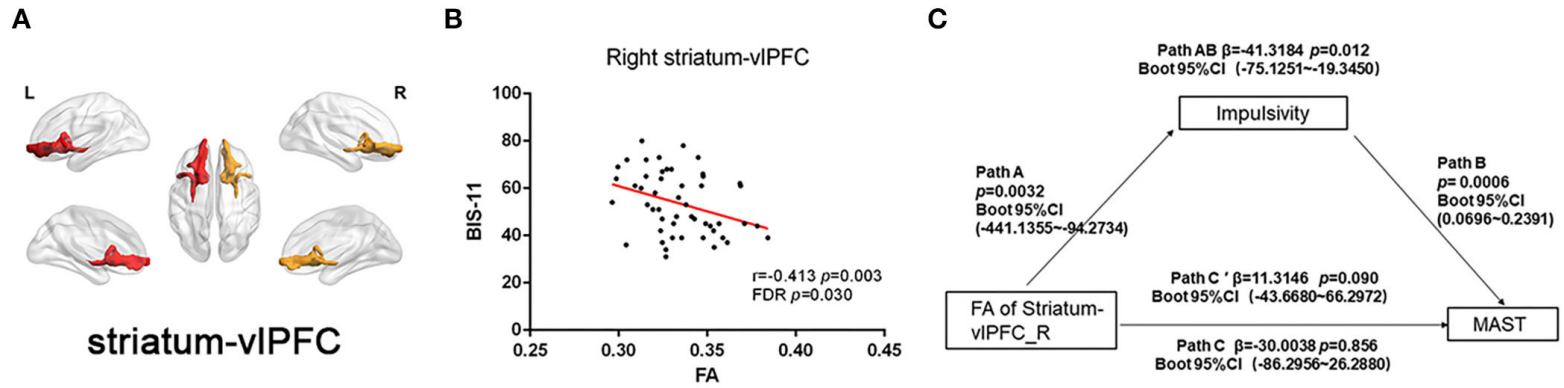
Correlation between FA of striatal circuits and impulsivity and mediation role. **(A)** Visualization of white matter connectivity target from the striatum to the ventrolateral prefrontal cortex; **(B)** Scatter plot with a significantly negative correlation between FA of right striatum–dorsolateral prefrontal cortex tract and BIS-11 score; and **(C)** Mediation analysis of FA of right striatum-vlPFC, impulsivity (BIS-11), and severity of alcohol dependence (MAST score).

Partial correlation was analyzed between FA of striatal circuits and BIS-11 in the fifty-one AD patients. The FA of the right striatum-vlPFC (*r* = 0.413, *p* = 0.003, FDR *p* = 0.030) and left striatum-vlPFC (r = −0.347, *p* = 0.015, FDR *p* = 0.075) was negatively associated with BIS-11 (Figure 4; Supplementary Table 5). However, there is no significant relationship between the FA of striatal circuits and BIS-11 in HC (Supplementary Table 6).

### Mediation analysis

We also performed a mediation analysis to test whether the association between reduced white matter integrity of the right striatum-vlPFC and severity of alcohol drinking (MAST score) could be mediated by impulsivity (BIS-11). We found the relationship between reduced FA of right striatum-vlPFC and MAST score was mediated by impulsivity [β = −41.3184 Boot 95% CI (−75.1251~−19.3450)] *p* = 0.012. However, the reduced FA could not directly predict the severity of alcohol use [β = −30.0038 Boot 95% CI (−86.2956~26.2880)] *p* = 0.856 (Figure 4). Considering the power to test the mediation analysis of right striatum-vlPFC, impulsivity, and AD severity, we calculated the *post-hoc* power through power Mediation package in R. The result of *post-hoc* power analysis indicated that the moderate sample size of the AD (*n* = 51) can achieve 0.75.

## Discussion

In our study, we found widespread abnormal white matter integrity of whole-brain skeleton and striatal circuits in AD compared with HC. We also found a negative relationship between FA of right striatum-vlPFC and BIS-11 in AD. Furtherly, the relationship between reduced FA of right striatum-vlPFC and severity of dependence was mediated by impulsive trait. Our study may provide the role of WM disruption of striatal circuits underlying the mechanism of impulsivity in AD.

In our study, we found mean alcohol intake per day was significantly positively correlated with impulsivity in AD. Our findings are consistent with some prior studies (9, 39). Higher impulsivity with the BIS-11 shows higher levels of cue reactivity than less impulsive drinkers (40). Impulsivity is generally described as a construct consisting of a predisposition toward rapid, unplanned reactions to internal or external stimuli without regard to the negative consequences (41). Thus, impulsivity has an influence on almost all stages of alcohol use and exacerbate disease progression (42, 43). Therefore, it is necessary to investigate the mechanisms of impulsivity to improve the prognosis of AD.

We found widespread WM skeleton microstructural differences in patients with AD compared with HC, mainly in the corpus callosum, frontal area, and association fibers, which were in agreement with two previous literatures (44, 45). Interestingly, a positive correlation was found between FA of forceps minor and BIS-11 in HC; however, there is no correlation found in AD. Impulsive traits are not necessarily pathological and likely reflect the desire/motivation to obtain high salience outcomes (46, 47). Thus, this may suggest higher WM integrity in the frontal portion of the corpus callosum may promote adaptive social behavior in HC. The forceps minor is a large fiber bundle that connects the bilateral prefrontal cortices (48), which play an important role in motor control (49). A recent study found WM reduced along the forceps major and forceps minor and the FA was negatively correlated with the impulsivity score in attention deficit hyperactivity disorder (50). Similar finding not found in AD in our study may attribute to the severe disruption effect of alcohol on callosum forceps.

We also found weaker WM integrity of striatal circuits in AD compared with HC, including left and right striatum–SMA, and left striatum–amygdala. Alcohol produces dysfunctions in functional connectivity between the striatum and other cortical areas (51, 52). Our DTI findings of decreasing microstructural connectivity of striatal circuits in AD performed a useful complement to previous work. As TBSS analysis fails to cover specific tracks between striatum and related ROIs, therefore, we selected ROIs associated with impulsive trait, including frontal cortex and the limbic basal ganglia. Specifically, the prefrontal cortex (dlPFC and vlPFC) has been linked to “top-down” cognitive control of inhibiting impulse that promotes risky behaviors and SMA has been related to the inhibition of motor impulse (53, 54). Striatum acts in concert with portions of the PFC to modulate impulsive behavior (55, 56). Insular cortex specifically outgoing projects to the striatum is also necessary for executive “top-down” control (57, 58). The amygdala receives input from midbrain dopamine neurons and innervates the striatum, which regulates risky reward seeking (59). The disrupted functional and structural connectivity of cortico-limbic-striatal systems may be the mechanism of impulsive reward seeking in AD.

In our study, a negative correlation was found between WM integrity of right striatum-vlPFC connection and BIS-11 in AD. The vlPFC has been proposed as a key area for inhibitory control of inappropriate impulse (60, 61). A recent study found AD displayed less modulation of activation in PFC when deciding to take risk decisions (62). Thus, diminished FA of striatum-vlPFC may presumably reflect ineffective PFC control over the striatum, which may account for disruption in inhibition control and impulsive behavior in AD. Our findings of abnormal microstructure of vlPFC to striatum were consistent with some functional MRI results (56). A previous study also found the reduced tract strength of striatum-vlPFC was associated with abstinence-induced increases in craving and the relapse in nicotine-dependent (37). Furthermore, we also found increased impulsivity mediated the correlation between lower FA of right striatum-vlPFC and the severity of alcohol dependence. This may suggest that WM disruption of certain cortico-striatal circuits could potentially reflect a vulnerability factor with increased impulsivity, which may promote severe alcohol consumption. It has been suggested that higher impulsivity may stem from the neurobiological effects of alcohol intake or, conversely, may be a premorbid deficiency of inhibitory control (13). However, more evidence is needed to address this causality relationship.

The strengths of this study were listed as follows. First, we explored the mechanisms of WM microstructures underlying impulsive trait in AD. Second, we addressed the WM integrity of striatal circuits by utilizing probabilistic tractography in AD to find the exact relationship with impulsive trait. However, there are also some limitations in our study. First, in spite that we examined the connectivity of the striatum circuits in AD, however, the segmentation of the striatum (e.g., ventral and dorsal striatum) may provide more information (10). Second, future studies with larger sample sizes could explore several other striatal tracts that might also be important for impulsive traits (63, 64). Finally, like most of clinical studies, our study was a cross-sectional study. We cannot directly address the causal relationship between impulsive trait and long-term alcohol dependence.

## Conclusion

In summary, we found abnormal white matter microstructure of striatal circuits in male AD patients. We also found a negative relationship between FA of right striatum-vlPFC and BIS-11 in AD. Meanwhile, the impulsive trait played a mediating effect between FA of striatum-vlPFC and severity of dependence. Thus, our findings could provide system-level insights into the abnormal integrity of striatal circuits in alcoholics and their potential roles as neuroimaging biomarkers for impulsive traits.

## Data availability statement

The raw data supporting the conclusions of this article will be made available by the authors, without undue reservation.

## Ethics statement

The studies involving human participants were reviewed and approved by Ethics Committee of Peking University Sixth Hospital. The patients/participants provided their written informed consent to participate in this study.

## Author contributions

HS and JY supervised the project, designed, edited, and led the experiments of this study. FW and PD collected the original clinical data. FW and GW conducted data analysis. FW prepared all the figures and tables, drafted the manuscript, and conducted analysis and interpretation of the results. JD, XG, ZN, and PL provided critical revision of the manuscript for important intellectual content. All authors contributed to the article and approved the submitted version.

## Funding

This work was supported by the National Natural Science Foundation of China (81971235, 81771429, and 82071552).

## Conflict of interest

The authors declare that the research was conducted in the absence of any commercial or financial relationships that could be construed as a potential conflict of interest.

## Publisher's note

All claims expressed in this article are solely those of the authors and do not necessarily represent those of their affiliated organizations, or those of the publisher, the editors and the reviewers. Any product that may be evaluated in this article, or claim that may be made by its manufacturer, is not guaranteed or endorsed by the publisher.
