# Erratum: Impulsivity trait mediates the relationship between white matter integrity of prefrontal–striatal circuits and the severity of dependence in alcoholism

**DOI:** 10.3389/fpsyt.2022.1107947

**Published:** 2022-12-06

**Authors:** Frontiers Production Office

**Affiliations:** Frontiers Media SA, Lausanne, Switzerland

**Keywords:** alcohol dependence, impulsivity, systematic striatal circuits, probabilistic tractography, diffusion tensor imaging

Due to a production error, the order of authors in the author list was incorrect. The correct author list is: Fei Wu^1^^†^, Ping Dong^1^^†^, Guowei Wu^2^, Jiahui Deng^1^, Zhaojun Ni^1^, Xuejiao Gao^1^, Peng Li^1^, Bing Li^1^, Junliang Yuan^3*^^†^and Hongqiang Sun^1^^*^^†^.

The publisher apologizes for this error and states that this does not change the scientific conclusions of the article in any way. The original article has been updated.

