# Supplementary material for: Impulsivity trait mediates the relationship between white matter integrity of prefrontal–striatal circuits and the severity of dependence in alcoholism
Source: Front Psychiatry. 2022 Sep 7;13:985948. doi: 10.3389/fpsyt.2022.985948 (PMC9490322; doi:10.3389/fpsyt.2022.985948)
Supplement: Supplementary file 1 [file Table_1.DOCX]

**Supplementary Table 1**. Correlations analysis between clinical and behavioral outcomes and BIS-11 in AD

|  | *r* | *p* | FDR *p* |
| --- | --- | --- | --- |
| C-VAS (post-pre) | 0.322 | 0.021 | 0.062 |
| AUQ (post-pre) | 0.302 | 0.031 | 0.062 |
| HR (post-pre) | -0.221 | 0.119 | 0.179 |
| SBP (post-pre) | -0.148 | 0.301 | 0.362 |
| age at first use | 0.002 | 0.988 | 0.988 |
| mean alcohol intake (g/d) | 0.353 | 0.011 | 0.042 |

Abbreviations: C-VAS (post-pre), visual analogue scales of craving change before and after alcohol cue exposure; AUQ (post-pre), the score of Alcohol Urge Questionnaire change before and after alcohol cue exposure; HR (post-pre), heart rate change before and after alcohol cue exposure; SBP (post-pre), systolic blood pressure change before and after alcohol cue exposure; Indexes indicate a statistically significant correlation with a *p*-value less than 0.05. *, indicated *p* < 0.05.

**Supplementary Table 2**. Difference whole WM skeleton between AD and HC

|  | White Matter Tracts | cluster | T | MNI coordinates of Peak | | | FDR *p* |
| --- | --- | --- | --- | --- | --- | --- | --- |
|  |  |  |  | X | Y | Z |  |
| FA | Forceps minor | 9862 | -6.36 | 95.1 | 125 | 94.4 | <0.001 |
|  | Forceps major | 706 | -4.73 | 62.6 | 74.6 | 93.3 | 0.004 |
|  | Superior longitudinal fasciculus L | 255 | -5.04 | 136 | 80.4 | 72.2 | 0.011 |
|  | Inferior fronto-occipital fasciculus R | 204 | -5.66 | 61.5 | 157 | 80.9 | 0.005 |
|  | Forceps minor | 200 | -5.53 | 105 | 175 | 63.6 | 0.004 |
|  | Superior longitudinal fasciculus L | 189 | -5.31 | 132 | 81.6 | 100 | 0.002 |
|  | Forceps major | 172 | -4.57 | 65.8 | 50 | 89.9 | 0.014 |
|  | Inferior fronto-occipital fasciculus R | 161 | -4.40 | 63.6 | 47.9 | 75.8 | 0.016 |
|  | Forceps minor | 118 | -4.27 | 71.9 | 178 | 78.2 | 0.015 |
|  | Inferior fronto-occipital fasciculus L | 110 | -5.60 | 127 | 116 | 58.5 | 0.004 |
|  | Superior longitudinal fasciculus L | 94 | -4.62 | 131 | 128 | 92 | 0.019 |
|  | Superior longitudinal fasciculus L | 81 | -5.25 | 128 | 71.4 | 104 | 0.008 |
|  | Forceps major | 63 | -4.24 | 111 | 39.6 | 72.3 | 0.016 |
|  | Anterior thalamic radiation L | 55 | -6.00 | 104 | 93.7 | 82.9 | 0.008 |
|  | Forceps minor | 48 | -4.76 | 76.4 | 175 | 58.6 | 0.018 |
|  | Corticospinal tract R | 44 | -4.78 | 71.5 | 112 | 66.1 | 0.023 |

Abbreviations: FA, fractional anisotropy; L, left; R, right. Indexes indicate a statistically significant difference between the two groups with a *p*-value less than 0.05.

**Supplementary Table 3**. Partial correlations analysis between FA of WM skeleton and BIS-11 in HC

Abbreviations: Barratt Impulsiveness Scale, BIS-11; L, left; R, right. Indexes indicate a statistically significant correlation with a *p*-value less than 0.05. *, **, and *** indicated differences at the *p* < 0.05, 0.01, and 0.001 values, respectively.

|  | White Matter Tracts | MNI coordinates of Peak | | | *r* | *p* | FDR *p* |
| --- | --- | --- | --- | --- | --- | --- | --- |
|  |  | X | Y | Z |  |  |  |
| FA | Forceps minor | 95.1 | 125 | 94.4 | 0.220 | 0.291 | 0.592 |
|  | Forceps major | 62.6 | 74.6 | 93.3 | -0.009 | 0.966 | 0.966 |
|  | Superior longitudinal fasciculus L | 136 | 80.4 | 72.2 | 0.191 | 0.360 | 0.592 |
|  | Inferior fronto-occipital fasciculus R | 61.5 | 157 | 80.9 | 0.047 | 0.822 | 0.939 |
|  | Forceps minor | 105 | 175 | 63.6 | 0.434 | 0.030 | 0.160 |
|  | Superior longitudinal fasciculus L | 132 | 81.6 | 100 | -0.017 | 0.936 | 0.966 |
|  | Forceps major | 65.8 | 50 | 89.9 | 0.187 | 0.370 | 0.592 |
|  | Inferior fronto-occipital fasciculus R | 63.6 | 47.9 | 75.8 | 0.094 | 0.653 | 0.816 |
|  | Forceps minor | 71.9 | 178 | 78.2 | 0.548 | 0.005 | 0.040* |
|  | Inferior fronto-occipital fasciculus L | 127 | 116 | 58.5 | 0.291 | 0.158 | 0.421 |
|  | Superior longitudinal fasciculus L | 131 | 128 | 92 | 0.373 | 0.067 | 0.268 |
|  | Superior longitudinal fasciculus L | 128 | 71.4 | 104 | -0.092 | 0.663 | 0.816 |
|  | Forceps major | 111 | 39.6 | 72.3 | 0.162 | 0.438 | 0.637 |
|  | Anterior thalamic radiation L | 104 | 93.7 | 82.9 | -0.210 | 0.313 | 0.592 |
|  | Forceps minor | 76.4 | 175 | 58.6 | 0.606 | 0.001 | 0.016* |
|  | Corticospinal tract R | 71.5 | 112 | 66.1 | 0.309 | 0.132 | 0.421 |

**Supplementary Table 4**. Partial correlations analysis between FA of WM skeleton and BIS-11 in AD

|  | White Matter Tracts | MNI coordinates of Peak | | | *r* | *p* | FDR *p* |
| --- | --- | --- | --- | --- | --- | --- | --- |
|  |  | X | Y | Z |  |  |  |
| FA | Forceps minor | 95.1 | 125 | 94.4 | -0.211 | 0.145 | 0.306 |
|  | Forceps major | 62.6 | 74.6 | 93.3 | -0.200 | 0.168 | 0.306 |
|  | Superior longitudinal fasciculus L | 136 | 80.4 | 72.2 | -0.309 | 0.031 | 0.216 |
|  | Inferior fronto-occipital fasciculus R | 61.5 | 157 | 80.9 | -0.219 | 0.131 | 0.305 |
|  | Forceps minor | 105 | 175 | 63.6 | -0.105 | 0.474 | 0.506 |
|  | Superior longitudinal fasciculus L | 132 | 81.6 | 100 | -0.198 | 0.172 | 0.305 |
|  | Forceps major | 65.8 | 50 | 89.9 | -0.245 | 0.089 | 0.285 |
|  | Inferior fronto-occipital fasciculus R | 63.6 | 47.9 | 75.8 | -0.161 | 0.269 | 0.331 |
|  | Forceps minor | 71.9 | 178 | 78.2 | -0.286 | 0.047 | 0.216 |
|  | Inferior fronto-occipital fasciculus L | 127 | 116 | 58.5 | -0.167 | 0.252 | 0.331 |
|  | Superior longitudinal fasciculus L | 131 | 128 | 92 | -0.277 | 0.054 | 0.216 |
|  | Superior longitudinal fasciculus L | 128 | 71.4 | 104 | -0.349 | 0.014 | 0.216 |
|  | Forceps major | 111 | 39.6 | 72.3 | -0.171 | 0.240 | 0.331 |
|  | Anterior thalamic radiation L | 104 | 93.7 | 82.9 | 0.062 | 0.672 | 0.672 |
|  | Forceps minor | 76.4 | 175 | 58.6 | 0.309 | 0.309 | 0.353 |
|  | Corticospinal tract R | 71.5 | 112 | 66.1 | 0.258 | 0.258 | 0.331 |

Abbreviations: Barratt Impulsiveness Scale, BIS-11; L, left; R, right. Indexes indicate a statistically significant correlation with a *p*-value less than 0.05.

**Supplementary Table 5**. Partial correlations analysis between FA of striatal circuits and BIS-11 in AD

| White Matter Tracts | *r* | *p* | FDR *p* |
| --- | --- | --- | --- |
| Striatum-Amygdala L | -0.018 | 0.901 | 0.901 |
| Striatum-Amygdala R | -0.234 | 0.105 | 0.210 |
| Striatum-vlPFC L | -0.347 | 0.015 | 0.075 |
| Striatum-vlPFC R | -0.413 | 0.003 | 0.030* |
| striatum-dlPFC L | -0.267 | 0.063 | 0.163 |
| striatum-dlPFC R | -0.265 | 0.065 | 0.163 |
| Striatum-SMA L | -0.187 | 0.198 | 0.283 |
| Striatum-SMA R | -0.044 | 0.764 | 0.849 |
| Striatum-Insula L | -0.215 | 0.137 | 0.228 |
| Striatum-Insula R | -0.162 | 0.267 | 0.334 |

Abbreviations: Indexes indicate a statistically significant correlation with a p-value less than 0.05. *, **, and *** indicated differences at the *p* < 0.05, 0.01, and 0.001 values, respectively.

**Supplementary Table 6**. Partial correlations analysis between FA of striatal circuits and BIS-11 in HC

| White Matter Tracts | *r* | *p* | FDR *p* |
| --- | --- | --- | --- |
| Striatum-Amygdala L | -0.059 | 0.780 | 0.963 |
| Striatum-Amygdala R | -0.160 | 0.446 | 0.643 |
| Striatum-vlPFC L | 0.337 | 0.099 | 0.450 |
| Striatum-vlPFC R | 0.158 | 0.450 | 0.643 |
| striatum-dlPFC L | 0.392 | 0.053 | 0.450 |
| striatum-dlPFC R | 0.266 | 0.199 | 0.498 |
| Striatum-SMA L | 0.307 | 0.135 | 0.450 |
| Striatum-SMA R | 0.166 | 0.428 | 0.643 |
| Striatum-Insula L | 0.008 | 0.970 | 0.970 |
| Striatum-Insula R | -0.035 | 0.867 | 0.963 |

Indexes indicate a statistically significant correlation with a p-value less than 0.05. *, **, and *** indicated differences at the p < 0.05, 0.01, and 0.001 values, respectively.
